# Precision Genome Engineering and Agriculture: Opportunities and Regulatory Challenges

**DOI:** 10.1371/journal.pbio.1001877

**Published:** 2014-06-10

**Authors:** Daniel F. Voytas, Caixia Gao

**Affiliations:** 1 Department of Genetics, Cell Biology, and Development and Center for Genome Engineering, University of Minnesota, Minneapolis, Minnesota, United States of America; 2 State Key Laboratory of Plant Cell and Chromosome Engineering, Institute of Genetics and Developmental Biology, Chinese Academy of Sciences, Beijing, China; Cornell University, United States of America

## Abstract

Plant agriculture is poised at a technological inflection point. Recent advances in genome engineering make it possible to precisely alter DNA sequences in living cells, providing unprecedented control over a plant's genetic material. Potential future crops derived through genome engineering include those that better withstand pests, that have enhanced nutritional value, and that are able to grow on marginal lands. In many instances, crops with such traits will be created by altering only a few nucleotides among the billions that comprise plant genomes. As such, and with the appropriate regulatory structures in place, crops created through genome engineering might prove to be more acceptable to the public than plants that carry foreign DNA in their genomes. Public perception and the performance of the engineered crop varieties will determine the extent to which this powerful technology contributes towards securing the world's food supply.

## Abstract 2

As Caixia Gao and Dan Voytas explain, new techniques make it possible to precisely edit plant genomic DNA, providing opportunities to create crop varieties that will help meet the challenges facing agriculture, including an expanding world population and environmental change.

This article is part of the *PLOS Biology* Collection “The Promise of Plant Translational Research.”

Over the past 100 years, technological advances have resulted in remarkable increases in agricultural productivity. Such advances include the production of hybrid plants and the use of the genes of the Green Revolution—genes that alter plant stature and thereby increase productivity [1],[2]. More recently, transgenesis, or the introduction of foreign DNA into plant genomes, has been a focus of crop improvement efforts. In the US, more than 90% of cultivated soybeans and corn contain one or more transgenes that provide traits such as resistance to insects or herbicides [3]. Transgenesis, however, has limitations: it is fundamentally a process of gene addition and does not harness a plant's native genetic repertoire to create traits of agricultural value. Furthermore, public concerns over the cultivation of crops with foreign DNA, particularly those generated by the introduction of genes from distantly related organisms, have impeded their widespread use. The regulatory frameworks created to protect the environment and to address public safety concerns have added considerably to the cost of transgenic crop production [4]. These costs have limited the use of transgenesis for creating crops with agriculturally valuable traits to a few high-profit crops, such as cotton, soybean, and corn.

The tools of genome engineering allow DNA in living cells to be precisely manipulated (reviewed in [5]). Although genome engineering can be used to add transgenes to specific locations in genomes, thereby offering an improvement over existing methods of transgenesis, a more powerful application is to modify genetic information to create new traits. Traditionally, new traits are introduced into cultivated varieties through breeding regimes that take advantage of existing natural genetic variation. Alternatively, new genetic variation is created through mutagenesis. With genome engineering, it is possible to first determine the DNA sequence modifications that are desired in the cultivated variety and then introduce this genetic variation precisely and rapidly. The ability to control the type of genetic variation introduced into crop plants promises to change the way new varieties are generated. Already genome engineering is being used in crop production pipelines in the developed world, and this technology can also be used to improve the crops that feed the burgeoning populations of developing countries.

## The Technological Underpinnings of Genome Engineering

Genome engineering is enabled by harnessing the cell's DNA repair pathways (reviewed in [5]). Most genome engineering techniques direct the repair of DNA double-strand-breaks (DSBs), which are introduced in the genome at or near the site where a DNA sequence modification is desired. Targeted DSBs are achieved using sequence-specific nucleases (SSNs)—enzymes that recognize and cleave the target locus with high specificity. The repair of the break can be directed to create a variety of targeted DNA sequence modifications, ranging from DNA deletions to the insertion of large arrays of transgenes.

**Figure 1:**
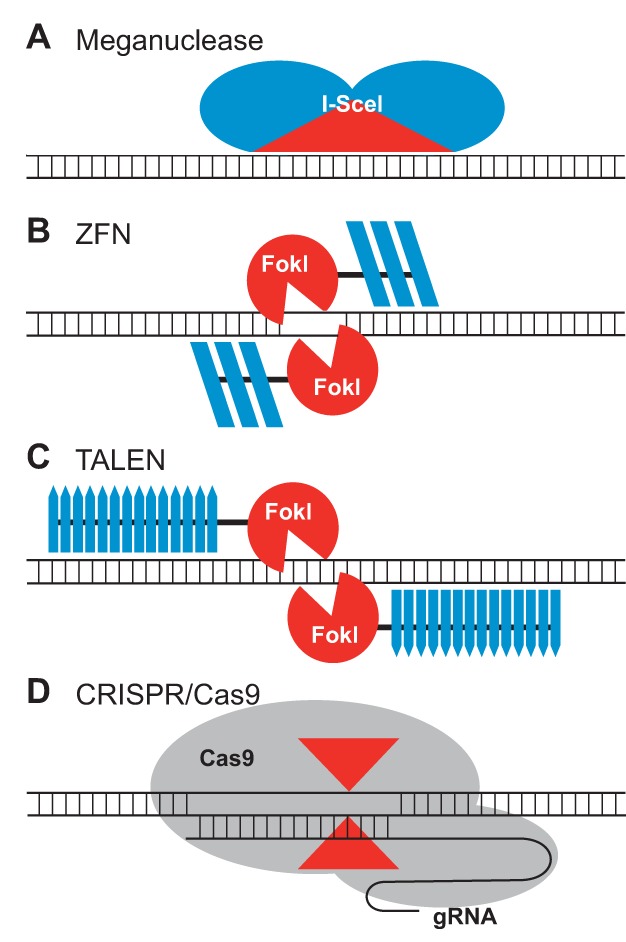
Schematics of the four classes of sequence-specific nucleases. (A) The meganuclease, I-SceI, is shown bound to its DNA target. The catalytic domain, which also determines DNA sequence specificity, is shown in red. (B) A ZFN dimer is illustrated bound to DNA. ZFN targets are bound by two zinc-finger DNA binding domains (dark blue) separated by a 5–7-bp spacer sequence. FokI cleavage occurs within the spacer. Each zinc finger typically recognizes 3 bp. (C) Depicted is a TALEN dimer bound to DNA. The DNA binding domains are in dark blue. The two TALEN target sites are typically separated by a 15–20-bp spacer sequence. Like ZFNs, the TAL effector repeat arrays are fused to FokI. Each TAL effector motif recognizes one base. (D) The CRISPR/Cas9 system recognizes DNA through base pairing between DNA sequences at the target site and a CRISPR-based guide RNA (gRNA). Cas9 has two nuclease domains (shown by red arrowheads) that each cleave one strand of double-stranded DNA.

There are currently four major classes of SSNs: engineered homing endonucleases or meganucleases, zinc finger nucleases (ZFNs), transcription activator-like effector nucleases (TALENs), and clustered regularly interspersed short palindromic repeats (CRISPR)/Cas9 reagents (Figure 1). Meganucleases are naturally occurring enzymes that bind and cleave large DNA sequence targets (from 12 to 40 bp) (Figure 1A) [6],[7]. Meganucleases can be engineered to recognize new sites; however, changes in target site specificity are difficult to achieve and often result in a reduction of catalytic activity; this has hindered their widespread use [7]. ZFNs, in contrast, bind DNA through an array of engineered zinc finger proteins, which are fused to the catalytic domain of the FokI endonuclease (Figure 1B) [8],[9]. FokI functions as a dimer, and so cleavage occurs when two ZFNs bind their targets and bring the FokI monomers into close proximity. TALENs are similar to ZFNs in that they have a DNA-binding domain fused to FokI; however, DNA recognition by TALENs is achieved through arrays of the TAL effector motif (Figure 1C) [10],[11]. The TAL effector motif is highly modular, and virtually any DNA sequence can be targeted with TALENs at high efficiency, making them easier to engineer than ZFNs. The most recent addition to the SSN arsenal is the CRISPR/Cas9 system. With CRISPR/Cas9, targeting is achieved through a guide RNA that base pairs with a specific chromosomal target sequence (Figure 1D) [12],[13]. The resulting RNA/DNA complex is then cleaved by the Cas9 nuclease. DNA targeting through base-pairing obviates the need to engineer a sequence-specific zinc finger or TAL effector array, and consequently, CRIPSR/Cas9 reagents are quickly emerging as the SSN of choice. Whereas the ability to engineer SSNs with the requisite DNA specificity was for a long time a bottleneck for genome engineering, DNA targeting can now be achieved with much greater efficiency. Certainly many challenges remain in terms of the delivery of genome engineering reagents to plant cells, but progress on this front is also advancing at a rapid pace [14],[15].

**Figure 2:**
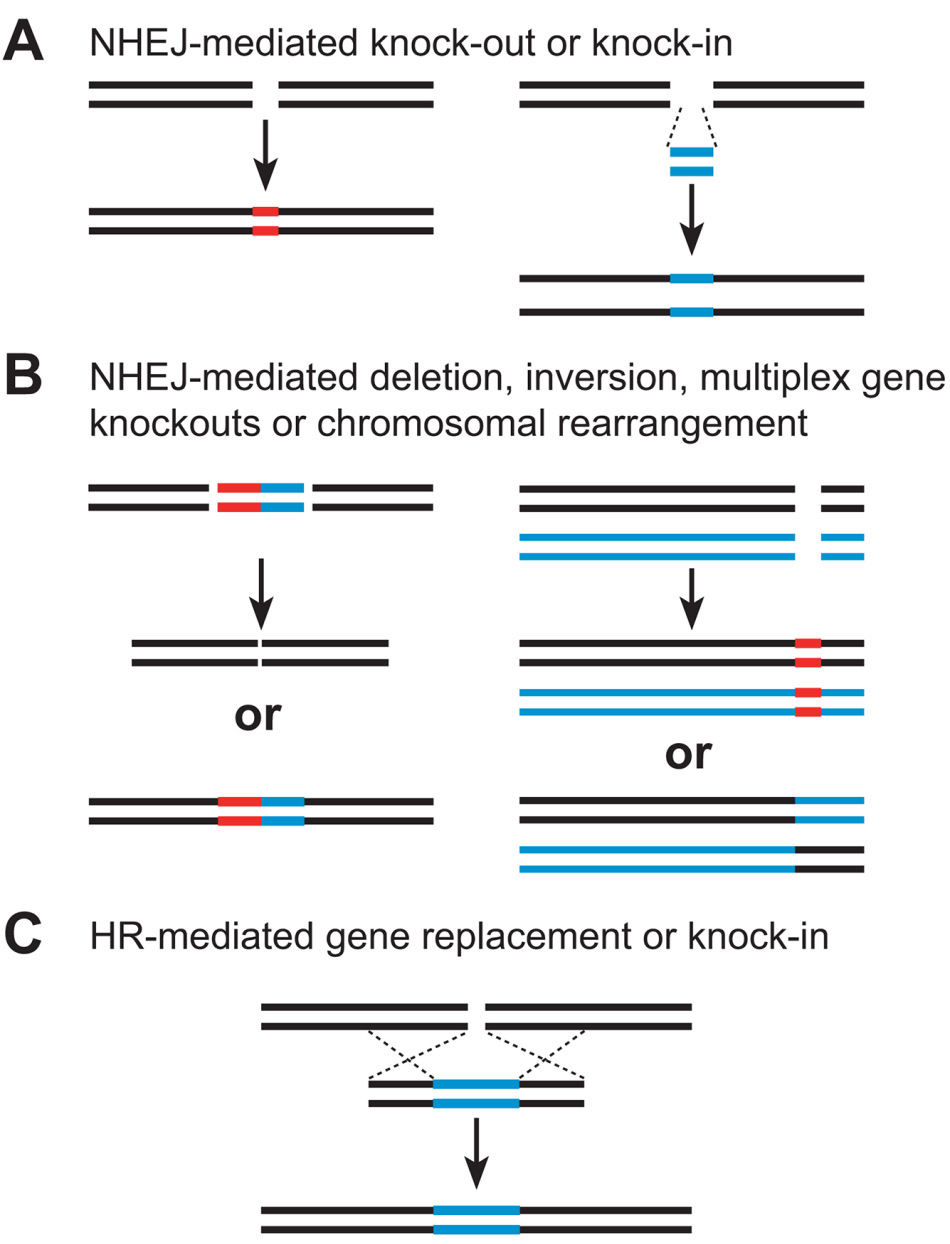
Targeted genome engineering by non-homologous end-joining or homologous recombination using sequence-specific nucleases. (A) NHEJ-mediated repair can result in small deletions or insertions at the target sites that can disrupt gene function (knock-outs, left). DNA fragments can be inserted via NHEJ-mediated ligation to create targeted insertions (knock-ins, right). (B) When two cuts are made by SSNs, NHEJ-mediated repair can result in either deletions or inversions of large genomic regions (left) or targeted gene deletions or chromosomal translocations (right). (C) HR-mediated repair, involving a homologous DNA template, leads to gene replacement or gene insertion.

How do targeted DSBs enable precise genome modifications? After breaks are introduced into the chromosome, one mechanism for break repair is non-homologous end-joining (NHEJ) (Figure 2A) [16],[17]. Although NHEJ is often precise, small deletions or more rarely insertions can be introduced at the junction of the newly rejoined chromosome. If the sequence modification causes a frameshift mutation or alters key amino acid residues in the target gene product, a knockout (loss of function) mutation can be created. Broken chromosome ends can also be joined to other DNA molecules that are introduced into the cell simultaneously with the SSN. The capture of heterologous DNA sequences can be used to achieve a targeted gene knock-in (targeted insertion) (Figure 2A). Finally, if two breaks are introduced into the chromosome simultaneously, targeted gene deletions or other rearrangements can result (Figure 2B). DNA repair through NHEJ is clearly a powerful means to achieve targeted DNA sequence modifications.

Homologous recombination (HR) is an alternative means to repair a broken chromosome. In HR, a repair template is used as a source of DNA sequence information that is copied to the broken chromosome to restore its integrity (Figure 2C) [18],[19]. HR can be harnessed to achieve targeted DNA sequence modifications by introducing into the cell both a SSN and a DNA repair template with sequence similarity to the break site (this process is referred to as gene targeting). Sequence variation that is carried by the repair template is copied by HR into the chromosome, thereby achieving targeted DNA sequence modification. Because the user specifies the type of sequence variation in the repair templates, HR offers numerous possibilities for manipulating plant genomes. For example, targeted gene knock-ins can be achieved using DNA repair templates with one or more transgenes flanked by sequences homologous to the target site. More subtle DNA sequence modifications can also be attained, including alterations to key amino acid residues within a gene's coding sequence, or changes in promoter elements or other cis-acting motifs that control gene expression. Thus, DNA repair by HR provides an unprecedented ability to manipulate a plant's genotype and consequently its phenotype.

## Opportunities for Crop Improvement through Genome Engineering

The promise of genome engineering for crop improvement is only just beginning to be realized. Because targeted modifications through NHEJ are relatively simple to achieve—requiring only expression of the SSN (Figure 2A)—most published examples of genome engineering in plants describe gene knockouts. Gene knockouts are valuable for studying gene function, because they link DNA sequences to phenotype. Some plant species important to the developing world such as cassava—a staple crop and key carbohydrate source for sub-Saharan Africa—lack extensive mutant resources. The generation of targeted gene knockouts through the use of SSNs should make genetic analyses in such orphan species possible. Many plants are polyploid or have undergone past episodes of polyploidy. Consequently, plant genomes typically have multiple, redundant genes and extensive gene family networks. SSNs can simultaneously create mutations in multiple gene family members [20],[21], enabling genetic analysis in often previously genetically intractable plants. Efforts to elucidate plant gene function will ultimately identify important genes that can be harnessed for crop improvement. To date, SSNs have been used to create gene knockouts in diverse plant species, including several important crops, such as barley [22], soybean [20], maize [23]–[28], rice [13],[29]–[35], wheat [13],[36], and sorghum [29].

In terms of trait development, the elimination of gene function through knockout mutations might seem to have limited utility. However, plants produce many products that negatively impact food quality, storage, and processing. Anti-nutritionals, such as glucosinolates, are produced by some plants to thwart insect pests, but they also have toxic effects when consumed by animals at high dose [37]. Cassava produces cyanide, which must be removed before human consumption [38]. One of the first targets for mutagenesis with SSNs was the maize gene encoding inositol-1,3,4,5,6-pentakisphosphate 2-kinase (*IPK1*), an enzyme that catalyzes the final step in phytate biosynthesis [27]. Phytate accounts for 75% of total phosphorus in maize seeds, and reducing phytate is of value because it is an anti-nutritional that limits mineral absorption and also contributes to environmental pollution through the waste stream.

Whereas gene knockouts can remove unwanted metabolites, they also make it possible to create plants that accumulate metabolites of value. For example, in the biosynthesis of fatty acids, loss of certain fatty acid desaturases in seeds allows monounsaturated fats to accumulate [39]. The resulting oils extracted from mutant seeds are healthier and have an improved shelf-life. Theoretically, the use of gene knockouts to disrupt biochemical pathways should make it possible to create plants that accumulate a variety of valuable biosynthetic intermediates.

An excellent example of how NHEJ can be used to create a trait of value has recently been demonstrated in rice [31]. The bacterial pathogen, *Xanthomonas oryzae*, is the causal agent of a blight that affects rice in both temperate and tropical climates and causes significant annual losses in rice productivity. During infection, the pathogen secretes effector proteins into rice cells that bind to the promoter region of the rice sucrose-efflux transporter gene (*OsSWEET14*). The binding of these effectors activates *OsSWEET14* transcription, which contributes to pathogen survival and virulence. TALENs were used to create a mutation in the effector binding site in the promoter of *OsSWEET14*, thereby eliminating the transcriptional induction of this gene by effectors and consequently reducing the pathogen's virulence.

Whereas plants with genomes altered via NHEJ are only just beginning to be reported, there are even fewer published examples of plants modified by HR. This is because HR is more challenging to implement [5]—chromosome cleavage by the nuclease must be coordinated with delivery of the DNA repair template (Figure 2C). Initial reports of HR in plants demonstrated the insertion of transgenes—principally marker genes—at precise chromosomal locations. Targeting transgene insertion to euchromatin should yield more predictable levels of transgene expression, and is therefore an improvement over the random integration achieved through traditional transgenesis. Furthermore, if multiple transgenes are inserted at the same site (stacked), then they will be transmitted in crosses as a single Mendelian locus. This greatly facilitates efforts to introduce numerous different transgenes into germplasm by breeding. Targeted gene insertion through HR has been demonstrated with different SSN platforms in several plant species, including tobacco [40], maize [27], and rice [13].

The real potential of HR for crop improvement, namely targeted gene replacement, remains to be fully realized. In one example, HR was used to introduce amino acid changes in a plant gene encoding acetolactate synthase (ALS)—an enzyme involved in branched-chain amino acid biosynthesis [40]. ALS is the target of several herbicides, including imidazolinones and sulfonylureas, and amino acid substitutions were introduced into the ALS gene that prevent inhibition of the enzyme by these herbicides. The resulting plants with the amino acid substitutions could grow in the presence of the herbicide. Commercially, herbicide tolerance is typically achieved through transgenesis, and one of the most widely used transgenes—the bacterial 5-enolpyruvylshikimate-3-phosphate synthase (EPSPS) gene—provides resistance to the herbicide glyphosate. Plants also encode EPSPS, and it should theoretically be possible to create glyphosate-tolerant plants by modifying the native gene in much the same way tolerance was created to ALS-inhibiting herbicides. The use of HR to create herbicide tolerance provides a nice point of comparison with transgenesis. In the long run, however, HR will be most effectively deployed for crop improvement when it is used to create traits that cannot be achieved through simple addition of a transgene.

## Regulating Crops with Edited Genomes

How will new crop varieties with precisely modified genomes be regulated? This question is currently being grappled with by regulatory authorities worldwide. The framework for making such decisions is the existing regulations covering transgenic crops, and different countries generally follow one of two approaches—process-based regulation considers whether biotechnology was used to create the crop, and product-based regulation considers attributes of the new variety itself (Box 1). The amount of regulation imposed upon crop varieties made through genome engineering will impact the cost of their development and how quickly they make it into the food supply. Hand in hand with regulation, the willingness of the public to accept food products made from genome engineered plants will also play a role in the extent to which this technology is fully used for crop improvement.

**Box 1. Regulatory Frameworks for New Crop Varieties**

**Process based regulatory frameworks** consider the techniques used to create new crop plant varieties. In general, if nucleic acids are introduced into plants or recombinant DNA technologies are deployed in the development of a crop, then regulation is triggered. The definitions and guidelines for regulation are based on those stipulated by organizations such as the United Nations Food and Agricultural Organization and treaties such as the Cartagena protocol. Process-based regulation is followed by the EU, Argentina, Brazil, and several other countries.

**Product-based regulatory frameworks** focus less on the technology used to develop the crop and more on the inherent risk of the final product; that is, the potential risk the new trait or attributes introduced into the plant poses to the public or the environment. The US and Canada use product-based regulation.

For crop varieties created by NHEJ, the issues under deliberation are somewhat straightforward. Mutations created with SSNs are often indistinguishable from those that occur naturally or that are created by conventional chemical, X- or gamma-ray mutagenesis. Furthermore, targeted modification can be achieved without incorporating foreign DNA into the plant's genome: mutagenesis using NHEJ is possible by transiently expressing the SSN within a plant cell (Box 2). Transient expression often results when SSN-encoding DNA constructs are delivered to cells. After expression of the SSN, the DNA constructs become degraded and are lost before becoming integrated into the plant's genome. Even if SSN DNA integrates into chromosomes, it is often unlinked to the site of modification and can be removed by crossing, leaving a non-transgenic plant line that carries only the desired DNA sequence change. Targeted mutagenesis can also be achieved by expressing SSNs transiently using viral vectors [14],[15], or by delivering them to cells as mRNA or protein. Protein delivery is particularly attractive from a regulatory standpoint, as the use of nucleic acids in product development is a key trigger for process-based regulation. Unlike nucleic acids, proteins are not transmitted from generation to generation, and so the delivery of SSNs as proteins is not much different from the use of standard mutagens.

**Box 2. Transgene-Free Methods to Create Mutant Plants with SSNs**

- Integration of SSN constructs into plant genomes. SSNs are expressed from the integrated DNA, and they act at a distal site. The SSN construct is then segregated away by crossing to obtain a plant with the mutation but no transgene.
- Transient delivery of SSNs to plant cells as DNA using Agrobacterium, biolistics, or protoplast transformation. SSNs are expressed from DNA constructs transiently before the DNA is degraded.
- Transient delivery of SSNs to plant cells as proteins or mRNAs. Since DNA is not delivered, no foreign DNA is incorporated into the plant genome.
- Transient delivery of SSNs using viral vectors. As viral vectors typically do not integrate into the plant genome, mutated plants are not transgenic.

Regulatory authorities have recently published preliminary opinions on the use of SSNs to create new crop varieties. In the US, the US Department of Agriculture (USDA) has recently stated that some NHEJ-induced mutations made by meganucleases and ZFNs fall outside their regulatory authority [41]; the USDA's position on the use of TALENs and CRISP/Cas9 reagents to engineer new plant traits is expected in the near future. To illustrate the consequence of such rulings, the ZFN-derived maize lines described above that have lower levels of phytate will not require the considerable body of data that typically must be assembled for regulatory approval and field planting [41]. Preparation of such regulatory packages is expensive (as much as US $35 million per transgenic event) and time consuming (taking up to 5.5 years to complete) [4]. The time and cost savings resulting from less regulation will be important factors in how quickly agricultural biotechnology companies adopt genome engineering. Reduced government regulation will also enable genome engineering to be applied to minor crops, such as vegetables or horticultural species, which lack the profit margins necessary to pay for governmental regulatory packages.

Other statements made recently by the USDA may impact certain approaches for mutant plant production. At least three recent rulings have indicated that the offspring of transgenic plant parents, which themselves lack a transgene, would not fall under the USDA's regulatory authority [42]. Thus, regulation may not pertain to mutant plants created by integrating an SSN construct in the genome and then segregating away the SSN to create transgene-free, mutant progeny.

Plants modified by HR warrant different consideration. Because a DNA template is required to copy information into a chromosomal locus, nucleic acids must necessarily be delivered to a plant cell to create the targeted modification. As mentioned above, for those countries that adhere to process-based regulation, this would likely trigger regulation of crop varieties made through HR. Consider, however, a situation where a plant gene is altered to have the same DNA sequence as an orthologous gene in another variety or species. Pathogen resistance, for example, might be achieved by identifying a disease resistance gene in a wild species and using it as a template in HR to replace a non-effective ortholog in a susceptible cultivar. Whereas such a cultivar could also be attained through conventional breeding, this is time-consuming. Furthermore, the resistance gene could have tight genetic linkage to unwanted loci that compromise fitness (so called linkage drag). It is our opinion that a different regulatory consideration should be given to cases where naturally occurring genetic variation is introduced.

For other types of genetic variation created by HR, it is likely that some threshold will have to be established as to the amount of variation that triggers regulation. Will it be the number of bases modified? The distribution of base changes over a certain length of DNA? Such distinctions seem rather arbitrary. It may be that plants engineered through HR will have to be considered on a case-by-case basis. Although determining the best means to regulate plants created by HR may prove challenging, such guidance is urgently needed.

The ability to modify genomes through HR makes it possible to generate crop varieties that have whole suites of genes altered to produce needed metabolites and to provide resistance to biotic and abiotic stresses. In plant synthetic biology, genome engineering through HR will likely become a cornerstone for this rapidly emerging field. Precise DNA sequence alterations will be important to realize opportunities in synthetic biology, such as increasing photosynthetic efficiency to attain higher yields (for example, engineering C3 plants, such as rice, to carry out more efficient C4 photosynthesis) [43]. Golden Rice, which has high levels of carotenoids in the grain, has been heralded as a means to relieve vitamin A deficiency in the developing world [44]. However, the transgenes that have been introduced to produce carotenoids in Golden Rice have generated resistance to its cultivation by those groups against transgenic food crops [45]. Could endogenous genes be manipulated by genome engineering to produce carotenoids in rice seed? Quite possibly; but would such plants receive a different public and regulatory reception?

Public acceptance will ultimately impact how widely genome engineering is deployed in agriculture: the public may simply not want products of genome engineering in the food supply, regardless of what different regulatory authorities decide. In Europe, for example, transgenic varieties are not cultivated, even those, such as Monsanto's YieldGard maize, which have received regulatory approval elsewhere. Similarly, Calgene's Flavr Savr tomato—engineered to remain firm after ripening—was approved in 1994 for release but failed in the supermarket for a variety of reasons including public acceptance [46]. As demands are placed on agriculture by an expanding world population, however, we may not always have the luxury of choice. We may need to deploy all resources to ensure global food security, including the many new crop varieties created through genome engineering that should greatly benefit both the consumer and the grower.

## Abbreviations

CRISPR: clustered regularly interspersed short palindromic repeats
HR: homologous recombination
NHEJ: non-homologous end-joining
SSN: sequence-specific nuclease
TALEN: transcription activator-like effector nuclease
ZFN: zinc-finger nuclease
